# Distinctive effect on nerve growth factor-induced PC12 cell neurite outgrowth by two unique neolignan enantiomers from *Illicium merrillianum*

**DOI:** 10.1038/srep16982

**Published:** 2015-11-20

**Authors:** Xinhui Tian, Rongcai Yue, Huawu Zeng, Honglin Li, Lei Shan, Weiwei He, Yunheng Shen, Weidong Zhang

**Affiliations:** 1 Department of Phytochemistry, School of Pharmacy, Second Military Medical University, 325 Guohe Road, Shanghai 200433, P. R. China; 2 School of Pharmacy, Shanghai Jiao Tong University, 800 Dongchuan Road, Shanghai 200240, P. R. China; 3 School of Pharmacy, East China University of Science and Technology, 130 Meilong Road, Shanghai 200237, P. R. China

## Abstract

Merrillianoid (**1**), a racemic neolignan possessing the characteristic benzo-2,7-dioxabicyclo[3.2.1]octane moiety, was isolated from the branches and leaves of *Illicium merrillianum*. Chiral separation of **1** gave two enantiomers (+)−**1** and (−)−**1**. The structure of **1** was established by comprehensive spectroscopic analysis and single crystal X-ray diffraction. The absolute configurations of enantiomers were determined by quantum mechanical calculation. Compound (+)−**1** exhibited a better neurotrophic activity than racemate **1** by promoting nerve growth factor (NGF) induced PC12 cell neurite outgrowth, while (−)−**1** showed a distinctive inhibitory effect. Furthermore, a mechanism study indicated that the two enantiomers influenced NGF-induced neurite outgrowth of PC12 cells possibly by interacting with the trkA receptor, and extracellular signal regulated kinases 1/2 (ERK1/2) and mitogen-activated protein kinase (MEK) in Ras/ERK signal cascade. But the phosphorylation level of serine/threonine kinase Akt1 and Akt2 in PI3K/Akt signal pathway showed no significant difference between (+)−**1** and (−)−**1**.

Nerve growth factor (NGF) is the first member of neurotrophin, it acts on sympathetic and sensory neurons and functions as a trophic factor for distinct populations of neurons in central nervous system^1^. The depletion of NGF has been linked with neurodegenerative disorders such as Alzheimer’s disease^2^, while over expression of it is related with other diseases such as epilepsy^3^, glioma^4^, tumor metastasis^5^,^6^ etc. Thus small-molecular neurotrophin agonist or antagonist is of high interest to promote or inhibit NGF activity. To find NGF agonist or antagonist activity compounds, we made use of rat pheochromocytoma-derived PC12 cells as a model system. In response to NGF, PC12 cells differentiate into sympathetic-like neurons and extend long neurites, providing a useful model for the investigation of neuronal differentiation, signaling and other neurobiological events^7^.

The functions of NGF are mediated by high-affinity tyrosine kinase receptor type 1 (TrkA) and the low-affinity non-enzymatic p75 neurotrophin receptor (P75^NTR^)^8^. Activation of trkA mediates neurite outgrowth and neuronal survival, whereas p75^NTR^ induces apoptosis in some neuron cells. Evidence suggests that NGF binding to TrkA leads to the activation of Ras/ERK and PI3K/Akt downstream pathways^9^. Since the two pathways are critical for NGF-induced neuronal differentiation and survival of PC12 cells^10^,^11^, we investigated the influence of compounds on the phosphorylation of associated proteins in Ras/ERK and PI3K/Akt pathway.

Natural products are rich sources of lead compounds for drug discovery due to their unique structure and bioactivities^12^. For example, neurotrophic 11-*O*-debenzoyltashironin^13^, (−)−hinokinin^14^,^15^ and merrilactone A^16^ with unique structure had been isolated from the pericarps of *Illicium merrillianum. I. merrillianum* is a shrub or small tree indigenous to southwestern China and Burma, and locally used as an antirheumatic agent^17^. In our continuous effort to find new bioactive natural products from *I. merrillianum*, we have previously reported a novel isopropyl (13 → 11)-*abeo*-9,11-*seco* abietane diterpenoid and six other abietane diterpenoids^18^.

In this study, merrillianoid (**1**), a racemic neolignan possessing unique benzo-2,7-dioxabicyclo[3.2.1]octane moiety was isolated from the branches and leaves of *I. merrillianum*, and chiral separation of **1** gave two enantiomers (+)−**1** and (−)−**1** (Fig. 1). Biological assay revealed that (+)−**1** had a better effect than racemate **1** in promoting NGF-induced PC12 cell neurite outgrowth at concentrations of 1–10 μM, while (−)−**1** showed quite distinctive inhibitory effect also at concentrations as low as 1 μM. Moreover, a mechanism study indicated that the two enantiomers exerted their promoting or inhibtory activities possibly by regulating the phosphorylation of TrkA receptor, the extracellular signal regulated kinases 1/2 (ERK1/2), and mitogen-activated protein kinase (MEK) in Ras/ERK pathway. However the phosphorylation level of serine/threonine kinase Akt1 and Akt2 in PI3K/Akt pathway showed no significant difference between (+)−**1** and (−)−**1**. These findings provided a pair of interesting neolignans for NGF-agonist and antagonist drug design, and revealed that absolute configurations dramatically affected biological activities of compounds.

## Results and Discussion

Compound **1** was obtained as colorless needle crystal. The IR spectrum displayed absorptions due to the C−O bands at 1033, 1203 cm^−1^, and the phenyl groups at 1464, 1508 cm^−1^. The HRESIMS spectrum gave a pseudo-molecular-ion peak [M + H]^+^ at *m/z* = 417.1915 (calcd. 417.1913), which corresponding to a molecular formula C_23_H_29_O_7_ with ten degrees of unsaturation.

The ^1^H NMR spectrum of **1** displayed four singlet aromatic protons at *δ*_H_ = 6.39, 6.45, 6.46, 6.86 ppm, and one methyl and five methoxyl groups at *δ*_H_ = 1.65, 3.55, 3.74, 3.77, 3.83, 3.85 ppm. Furthermore, the −CHCH_3_ fragment was identified owing to the doublet methyl resonance at *δ*_H_ = 1.36 ppm (d, 3H, *J* = 6.5 Hz) and the quartet proton resonance at *δ*_H_ = 4.49 ppm (q, 1H, *J* = 6.5 Hz). An interpretation of the ^13^C and DEPT NMR spectra of **1** revealed 22 signals attributable to 23 carbon atoms, including nine quaternary carbon atoms (*δ*_C_ = 152.3, 148.9, 147.9, 146.5, 143.1, 142.5, 117.9, 115.2, 106.0 ppm), seven methine groups (*δ*_C_ = 112.6, 110.9, 100.3, 96.8, 85.2, 47.9, 38.9 ppm), and seven methyl groups (*δ*_C_ = 56.4, 56.2, 56.1, 56.0, 56.0, 22.6, 20.7 ppm). The above ^1^H, ^13^C and DEPT NMR spectroscopic data suggested that there were two 1,2,4,5-tetrasubstituted phenyl groups in **1**, and some of the resonances were quite similar to those of lignanoids.

**Figure 1:**
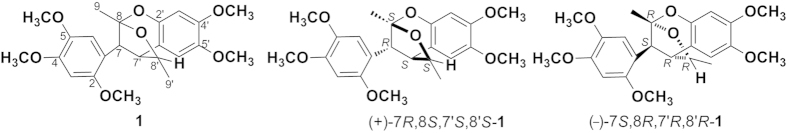
The chemical structures of 1, (+)−1 and (−)−1.

**Figure 2:**
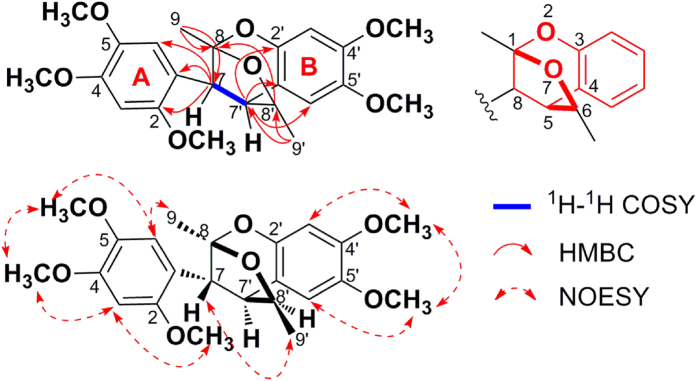
Key ^1^H−^1^H COSY, HMBC, and NOESY correlations of 1.

More detailed information about the structure of **1** came from the interpretation of ^1^H-^1^H COSY, HSQC, HMBC, and NOESY spectra (Fig. 2). The HMBC correlations from 2-OCH_3_ (*δ*_H_ = 3.83 ppm) to C-2 (*δ*_C_ = 147.9 ppm), 4-OCH_3_ (*δ*_H_ = 3.85 ppm) to C-4 (*δ*_C_ = 152.3 ppm), and 5-OCH_3_ (*δ*_H_ = 3.55 ppm) to C-5 (*δ*_C_ = 142.5 ppm), confirmed the primary locations of three methoxy groups. In the NOESY spectrum of **1**, the correlations between 3-H (*δ*_H_ = 6.46 ppm) and 2-OCH_3_, 4-OCH_3_, 6-H (*δ*_H_ = 6.86 ppm) and 5-OCH_3_, 4-OCH_3_ and 5-OCH_3_ were observed. The above NOESY correlations, together with the key HMBC correlations from 3-H and 6-H to C-1 (*δ*_C_ = 115.2), C-2, C-4, and C-5, confirmed the presence of the first 1,2,4,5-tetrasubstituted phenyl group in **1**. Furthermore the HMBC correlations of 7-H (*δ*_H_ = 3.82 ppm) with C-1, C-2, and C-6 (*δ*_C_ = 112.6 ppm), and 9-CH_3_ (*δ*_H_ = 1.65 ppm) with C-7 (*δ*_C_ = 38.9 ppm) and C-8 (*δ*_C_ = 106.0 ppm) assigned the C7-C8-C9 side chain to C-1, establishing phenyl propane unit **A**. The HMBC correlations from 4′-OCH_3_ (*δ*_H_ = 3.77 ppm) to C-4′ (*δ*_C_ = 148.9 ppm), and 5′-OCH_3_ (*δ*_H_ = 3.74 ppm) to C-5′ (*δ*_C_ = 143.1 ppm), confirmed the primary locations of another two methoxy groups. The NOESY correlations between 3′-H (*δ*_H_ = 6.45 ppm) and 4′-OCH_3_, 6′-H (*δ*_H_ = 6.39 ppm) and 5′-OCH_3_, 4′-OCH_3_ and 5′-OCH_3_ were observed, the above correlations, together with the HMBC correlations from 3′-H and 6′-H to C-1′ (*δ*_C_ = 117.9 ppm), C-2′ (*δ*_C_ = 146.5 ppm), C-4′ and C-5′ confirmed the presence of another 1′,2′,4′,5′-tetrasubstituted phenyl group in **1**. The HMBC correlations of 7′-H (*δ*_H_ = 3.03 ppm) with C-1′, C-2′, and C-6′ (*δ*_C_ = 110.9 ppm), and 9′-CH_3_ (*δ*_H_ = 1.36 ppm) with C-7′ (*δ*_C_ = 47.9 ppm) and C-8′ (*δ*_C_ = 85.2 ppm) assigned the C7′-C8′-C9′ side chain to C-1′, and established phenyl propane unit **B**. A spin coupling system H-7/H-7′ was established by the ^1^H-^1^H COSY spectrum, which indicated that phenyl propane units **A** and **B** were connected by C(7′)-C(7) bond. The HMBC cross-peak from 8′-H (*δ*_H_ = 4.49 ppm) to C-8, suggesting the existence of an ether bridge between C-8′ and C-8, forming a tetrahydrofuran ring. Deducting nine degrees of unsaturation accounted for two 1,2,4,5-tetrasubstituted phenyl groups and one tetrahydrofuran ring, the remaining one degree of unsaturation suggested that an additional ring was required. On the basis of the chemical shift, C-8 was supposed to link to C-2′ through an oxygen atom. Thus, the structure of **1** was established as depicted, and named merrillianoid, which possessed unique structure featured with a ketal formed between C-8 and C-2′, C-8′, forming a characteristic benzo-2,7-dioxabicyclo[3.2.1]octane moiety (Fig. 1). The proposed structure was further supported by the fragment ions at *m/z* 439 [M + Na]^+^, 247, 203 in the MS, MS^2^, MS^3^ spectra of **1** (see supporting information Fig. S1, S2).

**Figure 3:**
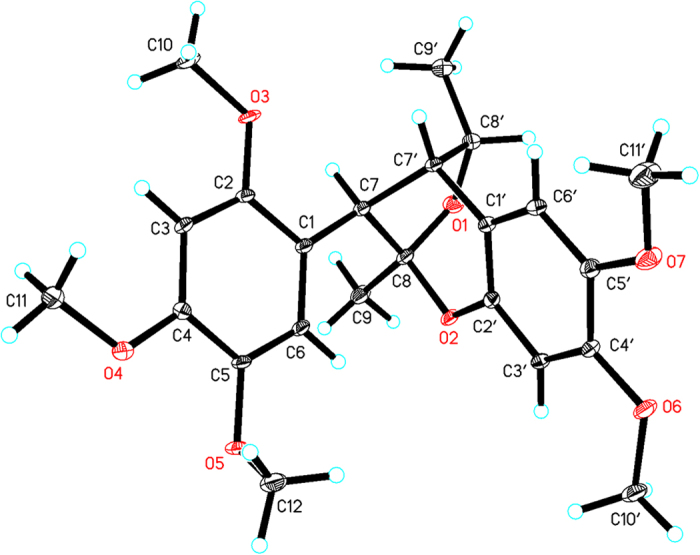
Single crystal X-ray diffraction of 1.

In the NOESY spectrum of merrillianoid (**1**), the correlations of 9′-CH_3_ with 7-H, and 9-CH_3_ with 6-H established the relative configuration of **1**. The single crystal X-ray diffraction (Cu-K*α* radiation; CCDC number: 1055836) further confirmed the relative configuration of **1** (Fig. 3). However, the space group *P*2_1_/c of **1** was achiral, which was an indicative of its racemic nature. The lack of optical activity furtherly confirmed that **1** was racemic. Subsequent HPLC separation of **1** on a chiral column led to two enantiomers (+)−**1** and (−)−**1**, which had opposite optical rotation and Cotton effects (see supporting information Fig. S7, S8). However, the single crystals of either of the two optically pure enantiomers can not be obtained in several solvent systems.

**Figure 4:**
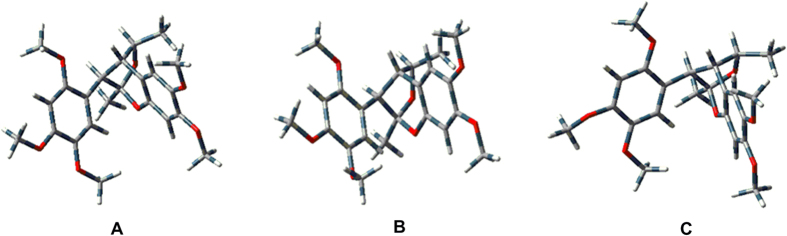
Optimized geometries of (+)−1 (A–C) at the B3LYP/3-21G level.

**Figure 5:**
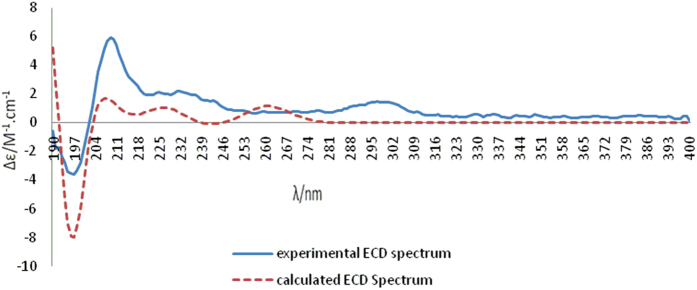
Experimental CD spectrum of (+)−1 and the calculated ECD spectrum of (7*R*,8*S*,7′*S*,8′*S*)−1 in methanol at the B3LYP/3-21G level with the PCM model.

Recently, quantum mechanical computation of electronic circular dichroism (ECD) has been successfully applied to determine the absolute configurations of natural products^19^,^20^. To determine the absolute configurations of the two enantiomers, we compared the experimentally observed CD spectra of (+)−**1** and (−)−**1** with the calculated ECD spectra obtained by quatum mechanical computation with the Gaussian03 software (Revision E.03. [Gaussian, Inc. (ed)] (Wallingford CT, USA, 2004)). Because of the rigid structure of **1** and the relative configuration established by NOESY experiment and single crystal X-ray diffraction, the arbitrarily assigned absolute configuration of 7*R*,8*S*,7′*S*,8′*S* for (+)−**1** was geometrically optimized by using density functional theory (DFT) at B3LYP/3-21G level to afford three preffered conformers **A**–**C** (ΔE < 5.0 kJ/mol) (Fig. 4). The ECD spectra of conformers **A**–**C** was then calculated at the B3LYP/3-21G level in the methanol solution with the PCM model. Each calculated ECD spectrum was assigned a Boltzmann weight according to the energy of the minimized conformers at 298.15 K and overlaid to give the calculated ECD spectrum. As depicted in Fig. 5, the calculated ECD spectrum for (7*R*,8*S*,7′*S*,8′*S*)-**1** matched well with the experimentally measured CD spectrum of (+)−**1**, which showed negative Cotton effects at 190–200 nm, and positive Cotton effects at 204–218 nm. Thus the absolute configuration of (+)−**1** was assigned as 7*R*,8*S*,7′*S*,8′*S*. Similarly, the absolute configuration of (−)−**1** was determined to be 7*S*,8*R*,7′*R*,8′*R* by the same method as (+)−**1** (see supporting information Fig. S6, S8).

**Figure 6:**
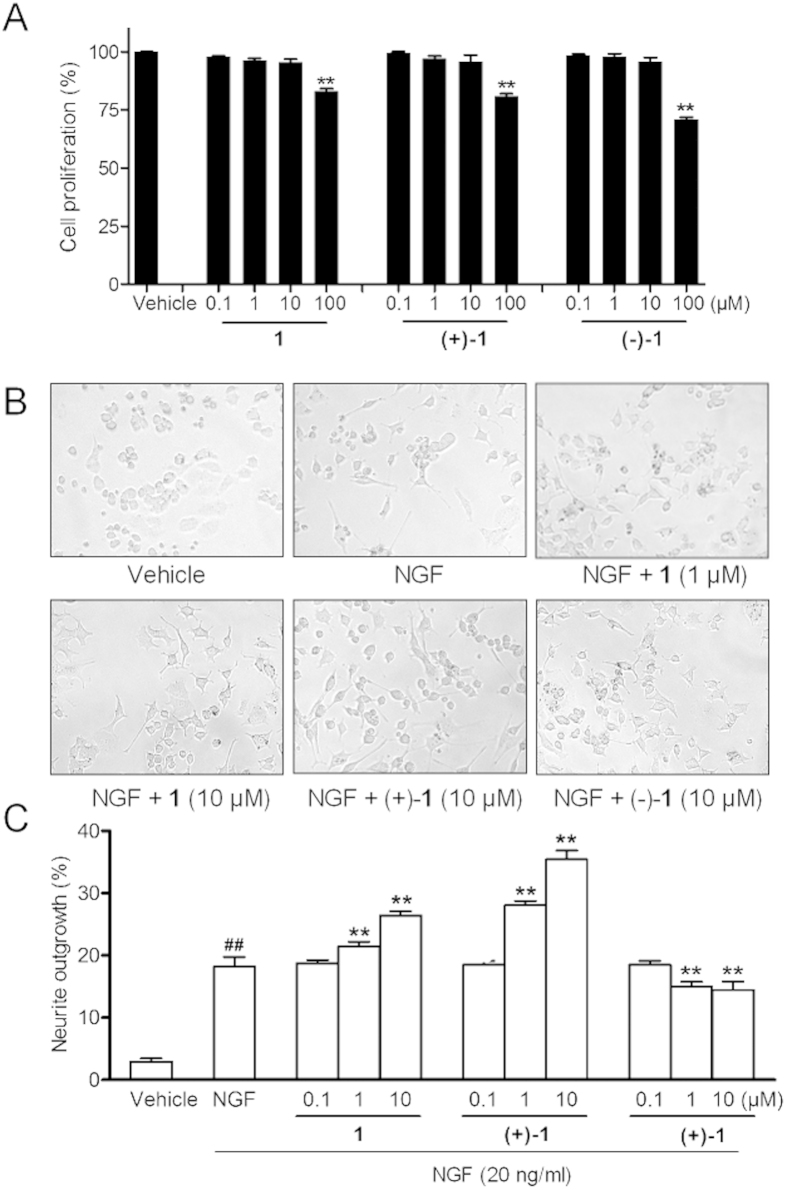
(**A**) PC12 cells proliferation after exposure to 0.1% DMSO or serial concentrations of **1**, (+)−**1** and (−)−**1**, for 24 h. (**B**) Representative images of the effects of **1**, (+)−**1**, and (−)−**1** on NGF-induced neurite outgrowth of PC12 cells. (**C**) Effects of **1**, (+)−**1**, and (−)−**1** on NGF-induced neurite outgrowth of PC12 cells at 0.1, 1, and 10 μM (Values were considered as significant at: ^##^*P* < 0.01 vs. vehicle, **P < 0.01 vs. 20 ng/mL NGF).

The NGF-mediated neurotrophic activity of enantiomers (+)−**1** and (−)−**1**, and racemate **1** were evaluated using rat pheochromocytoma (PC12) cells as a model system of neuronal differentiation^21^. As shown in Fig. 6A, all compounds at concentrations of 0.1–10 μM had no cytotoxic activity against PC12 cells by MTT assay. Then we further studied their effects on neurite outgrowth of PC12 cells (Fig. 6B,C). The mean value was expressed as percent of neurite-bearing cells in samples treated with compounds with or without 20 ng/mL NGF. The results showed that all compounds didn′t morphologically affect neurite outgrowth of PC12 cells in the absence of NGF. However, when in the presence of 20 ng/mL NGF, racemate **1** significantly increased PC12 cell differentiation rates (21.48 ± 0.71% at 1 μM, 26.40 ± 0.63% at 10 μM, *P* < 0.01), comparing with 20 ng/mL NGF alone (18.22 ± 1.53%, *P* < 0.01). Enantiomer (+)−**1** had a better promoting effect than racemate **1** with differentiation rates of 28.05 ± 0.71% at 1 μM, 35.47 ± 1.40% at 10 μM (*P* < 0.01), while (−)−**1** exhibited a distinctive inhibitory effect with neuronal differentiation rates of 17.05 ± 0.71% at 1 μM, and 14.47 ± 1.40% at 10 μM (*P* < 0.01). Overall, despite having the same planar structure, the opposite absolute configurations for (+)−**1** and (−)−**1** resulted in distinctive effects on NGF-induced PC12 cell differentiation.

**Figure 7:**
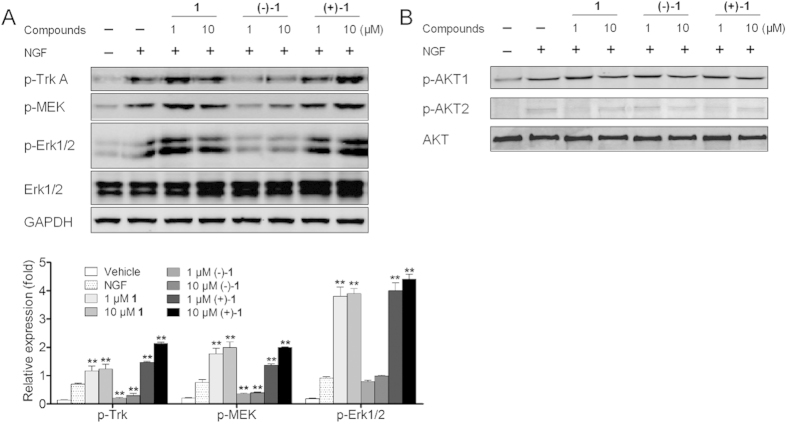
Western blotting analysis after 30 min treatment of PC12 cells with 1, (+)−1, and (−)−1 in NGF-induced TrkA, MEK, ERK1/2, Akt1, and Akt2 phosphorylation as indicated. Data shown are the results of three different experiments and are represented as the relative densities of protein bands normalized to GAPDH (**P < 0.01 vs. vehicle).

We further investigated the possible molecular mechanism of merrillianoids on NGF-induced PC12 cell differentiation by western blotting analysis^22^. Since trkA receptor activation leads to the phosphorylation of extracellular signal-regulated protein kinase 1/2 (ERK1/2) via mitogen-activated protein kinase (MEK) in the process of neurite outgrowth of PC12 cells^23^, we investigated the influences of these compounds on the phosphorylation of TrkA, MEK and ERK1/2 during the NGF-induced neurite outgrowth of PC12 cells (Fig. 7A). Treatment of PC12 cells with NGF (20 ng/mL) induced the phosphorylation of TrkA, MEK and ERK1/2 for 30 min. The relative phosphorylation level of TrkA, MEK and ERK1/2 of **1** and (+)−**1** both increased significantly comparing to 20 ng/mL NGF (0.70 ± 0.037 for TrkA, 0.76 ± 0.107 for MEK, 0.92 ± 0.048 for ERK1/2), and the phosphorylation level of (+)−**1** at 10 μM (2.13 ± 0.052 for TrkA, 1.99 ± 0.030 for MEK, 4.40 ± 0.179 for ERK1/2) was higher than that of racemate **1** (1.23 ± 0.174 for TrkA, 1.90 ± 0.202 for MEK, 3.90 ± 0.052 for ERK1/2). The phosphorylation level of (+)−**1** and **1** was in proportion to their neurite outgrowth promoting activities. As for (−)−**1**, the relative ratio of p-TrkA, and p-MEK decreased dramatically (0.30 ± 0.074 for TrkA, 0.40 ± 0.020 for MEK at 10 μM) comparing with 20 ng/mL NGF. In a word, compound (+)−**1** promoted the NGF-induced neurite outgrowth of PC12 cells by enhancing the phosphorylation level of TrkA receptor, and MEK, ERK1/2 in Ras/ERK signaling pathway, while (−)−**1** inhibited neurite outgrowth of PC12 cells via suppressing the phosphorylation of the above proteins.

The PI3K/Akt signaling cascade regulated cell survival and suppress apoptosis in a wide range of neuronal cell types^10^,^24^,^25^. Thus we detected the influence of all compounds on the phosphorylation of Akt-related serine/threonine kinase Akt1 and Akt2 during the NGF-induced PC12 cell differentiation. The western blotting analysis showed that there was no significant difference between all trial groups in the phosphorylation level of Akt1 and Akt2 (Fig. 7B).

In conclusion, two neolignan enantiomers (+)−**1** and (−)−**1** with characteristic benzo-2,7-dioxabicyclo[3.2.1]octane moiety were isolated from the branches and leaves of *I. merrillianum*. The kind of neolignans with benzo-2,7-dioxabicyclo[3.2.1]octane bridged-ring was first encountered in nature. Moreover (+)−**1** was found to have a better effect than racemate **1** in promoting NGF-induced neurite outgrowth of PC12 cells possibly by increasing the phosphorylation level of TrkA receptor, and MEK, ERK1/2 in Ras/ERK signaling cascade at concentrations of 1–10 μM, while its enantiomer (−)−**1** played the opposite role at the same concentrations. Opposite absolute configuration leads to big difference between the interaction of compounds and the PC12 cells. However the phosphorylation level of Akt1 and Akt2 in the PI3K/Akt pathway showed no significant differences between (+)−**1** and (−)−**1**. The results reported in this paper offer a pair of interesting neolignan enantiomers for potential NGF agonist and antagonist drug development.

## Materials and Methods

### General

1D and 2D NMR spectra were recorded on a Bruker Ascend 500 NMR spectrometer with TMS as internal standard; ESIMS, MS^2^, and MS^3^ were measured on the Agilent LC/MSD Trap XCT spectrometer and HRESIMS were performed on an Agilent 6520 Accurate-MS Q-TOF LC/MS system; Optical rotations were obtained with an Autopol VI (Rudolph Research Analytical, Hackettstown, NJ); CD spectra were recorded on a Chirascan spectrometer (Applied Photophysics, UK); IR spectrum was acquired with a Bruker Vector-22 spectrometer with KBr pellets; Colum chromatography was performed on silica gel (80–100 mesh, Huiyou Silica Gel Development Co., Ltd. Yantai, China); Reversed phase medium pressure liquid chromatography (RP-MPLC) was performed on a Buchi Sepacore system; TLC analysis was run on HSGF_254_ silica gel plates (1–40 μm, Huiyou Silica Gel Development Co., Ltd. Yantai, China); Preparative TLC was conducted with pre-coated silica gel HSGF_254_ plate (Huiyou Silica Gel Development Co., Ltd. Yantai, China).

### Plant Material

The branches and leaves of *I. merrillianum* were collected in Gongshan county, Yunnan province, P. R. China, in August 2011, and authenticated by Prof. Han-Ming Zhang of Second Military Medical University. A voucher specimen (No. 20110815) is deposited in the School of Pharmacy, Second Military Medical University.

### Extraction and Isolation

The air-dried and chopped branches and leaves of *I. merrillianum* (20 kg) were extracted with 95% EtOH (3 × 80 L) for three times (3 × 1 hr) to afford a crude extract (1.7 kg) after removal of solvent under low pressure. The extract was suspended in water and partitioned with petroleum ether (488 g), EtOAc (836 g), and *n*-BuOH (526 g) successively. The petroleum ether extract was subjected to silica gel column chromatography (CC) (3.4 kg, 80–100 mesh), using petroleum ether-EtOAc gradient elution (50:1–0:1) to afford seven fractions A (51 g), B (86 g), C (95 g), D (16 g), E (21 g), F (129 g), G (17 g). Fraction F was subjected to RP-MPLC (MeOH/H_2_O, 40–100%) to give five fractions F-1 (30 g), F-2 (21 g), F-3 (15 g), F-4 (31 g), F-5 (13 g). Fraction F-5 was further subjected to RP-MPLC (MeOH-H_2_O, 40–100%) to give five fractions (F-51, F-52, F-53, F-54, F-55). Compound **1** (14.8 mg) was isolated from fraction F-53 by applying preparative TLC using petroleum ether-Me_2_O (3:1, R_*f*_ = 0.43) developer.

### Chiral seperation of 1

HPLC chiral separation of **1** was performed on a Chiralpak IA column (USA): 0.46 cm I.D. × 25 cm; Flow rate: 1.0 mL/min; Solvent: Hexane/DCM/DEA = 45/55/0.1 (v/v/v); Temperature: 30 °C; Wavelength: 254 nm; Injection volume: 10 μL for each sample; The retention time of (+)−**1** (4.0 mg) and (−)−**1** (5.0 mg) was 7.935 and 8.635 min, respectively, and the peak area ratio of (+)−**1** to (−)−**1** was 0.8:1 (Fig. S4).

### Merrillianoid (1)

colorless needle crystal (MeOH); mp. 58 ∼ 59 °C; [α]25 D of (+)−**1**: + 48.0 (*c* 0.25, MeOH), [α]25 D of (−)−**1**: −48.0 (*c* 0.25, MeOH); CD of (+)−**1** (*c* 1.00 mmol/L, CH_3_OH, 25 °C) nm (Δε) 197 (−3.5), 210 (+5.8), CD of (−)−**1** (*c* 1.00 mmol/L, CH_3_OH, 25 °C) nm (Δε) 197 (+3.5), 210 (−5.8); IR (KBr) *ν*_max_ 2935, 1618, 1508, 1465, 1204, 1034, 884 cm^−1^; HRESIMS *m/z* 417.1915 [M + H]^+^ (calcd. for C_23_H_29_O_7_, 417.1913); ^1^H-NMR (500 MHz, CDCl_3_) *δ*: 1.36 (3H, d, *J* = 6.5 Hz, H-9′), 1.65 (3H, s, H-9), 3.03 (1H, d, *J* = 3.5 Hz, H-7′), 3.55 (3H, s, OCH_3_-5), 3.74 (3H, s, OCH_3_-5′), 3.77 (3H, s, OCH_3_-4′), 3.82 (1H, m, H-7), 3.83 (3H, s, OCH_3_-2), 3.85 (3H, s, OCH_3_-4), 4.49 (1H, q, *J* = 6.5 Hz, H-8′), 6.39 (1H, s, H-6′), 6.45 (1H, s, H-3′), 6.46 (1H, s, H-3), 6.86 (1H, s, H-6); ^13^C-NMR (125 MHz, CDCl_3_) *δ*: 115.2 (C-1), 147.9 (C-2), 96.8 (C-3), 152.3 (C-4), 142.5 (C-5), 112.6 (C-6), 38.9 (C-7), 106.0 (C-8), 22.6 (C-9), 117.9 (C-1′), 146.5 (C-2′), 100.3 (C-3′), 148.9 (C-4′), 143.1 (C-5′), 110.9 (C-6′), 47.9 (C-7′), 85.2 (C-8′), 20.7 (C-9′), 56.1 (OCH_3_-2), 56.2 (OCH_3_-4), 56.0 (OCH_3_-4′, 5), 56.4 (OCH_3_-5′).

### Computational Details

The theoretical calculations of (+)−**1** and (−)−**1** were performed on an IBM cluster machine using Gaussian03 package. Conformational analysis for 7*R*,8*S*,7′*S*,8′*S*-**1** and 7*S*,8*R*,7′*R*,8′*R*-**1** was initially carried out via the Molecular Operating Environment (MOE) software package using the MMFF94 force field. The obtained conformers were further optimized, and verified as the true minima of potential energy surface using TDDFT at B3LYP/3-21G(d) basis set level. Conductor-like polarizable continuum model (CPCM) was adopted to consider solvent effects using the dielectric constant of methanol (*ε* = 32.6). The 21 lowest electronic transitions were calculated and the rotational strengths of each electronic excitation were given using both dipole length and dipole velocity representations. ECD spectra were stimulated using a Gaussian function with a half-bandwidth of 0.3 eV. Equilibrium populations of conformers at 298.15 K were calculated from their relative free energies (∆G) using Boltzmann statistics. The overall ECD spectra of (+)−**1** and (−)−**1** were then generated according to Boltzmann weighting of each conformers.

### Conformational analysis

Conformational analysis was initially carried out using Maestro7.5 conformational searching, together with the MMFF94 molecular mechanics methods. MMFF94 structures were reoptimized using ab initio DFT at the B3LYP/3-21G (d) level. Energy of stable conformations were calculated and led to the relative energy, which in turn allowed the room temperature equilibrium populations to be calculated according to Maxwell - Boltzmann distribution law (Eqn 1).


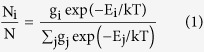


### Original ECD data calculations

Excitation energy (in nm) and rotatory strength R (velocity form R^vel^ and length form R^len^ in 10^–40^ erg-esu-cm/Gauss) between different states were calculated by time dependant density functional theory (TDDFT) at B3LYP/3-21G (d)//B3LYP/3-21G (d) level in methanol solution. All calculations are performed by the Gaussian03 program package.

### ECD simulation

The ECD spectra were then simulated by overlapping Gaussian functions for each transition according to Eqn 2:


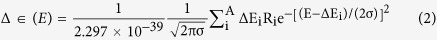


where σ is the width of the band at 1/e height and Δ*Ei* and *Ri* are the excitation energies and rotatory strengths for transition *i*, respectively, σ = 0.30 eV and R^vel^ have been used in this work. Conformational analysis has been carried out and theoretically weighted ECD spectra have been simulated at different levels mentioned above.

### Chemicals and Reagents for Biological activities

The undifferentiated rat pheochromocytoma cells line (PC12) were obtained from the Cell Bank of Shanghai Institute of Biochemistry & Cell Biology, Shanghai Institute for Biological Sciences, Chinese Academy of Sciences; NGF was purchased from Wuhan Hiteck Biological Pharma Co., Ltd. China; Dimethylsulfoxide (DMSO) and [3-(4,5-dimethylthiazol-2-yl)-2,5-diphenyltetrazolium bromide] (MTT) were purchased from Sigma (St. Louis, Mo., USA); Dulbecco’s Modified Eagle’s Medium (DMEM), a penicillin/streptomycin mixture, and fetal calf serum (FCS) and horse serum (HS) were purchased from Gibco (Grand Island, New York); Antibodies for p-TrkA, p-MEK, p-ERK1/2, p-Akt1 (Ser473), p-Akt2 (Thr308) and ERK1/2, Akt were obtained from Cell Signaling Technology (Beverly, MA); Anti-mouse and anti-rabbit secondary antibody were purchased from Odyssey (Li-Cor Biosciences, Lincoln, NE, USA); GAPDH (glyceraldehyde-3-phosphate dehydrogenase) was purchased from Beyotime Biotechnology (Jiangsu, China).

### MTT assay

The assay was performed in triplicate. PC12 cells were maintained in DMEM containing 5% FCS and 10% HS supplemented with 100 U/mL penicillin and 100 μg/mL streptomycin in a humidified atmosphere containing 5% CO_2_ at 37 °C. The tested compounds were dissolved in DMSO, and the final volume for DMSO in each well was no more than 0.1%. To rule out a potential influence of the solvent, we conducted a corresponding control experiments. PC12 cells were seeded in 96-well plate at a density of 5 × 10^4^/well. After cell attachment overnight, each well was treated with different concentrations of test compounds or 0.1% DMSO for 24 h. Then 10 μL of MTT solution (5 mg/mL) was added to each well and the cultures were incubated for another 4 h at 37 °C. The supernatant was then removed and 100 μL of DMSO was added to each well and agitated at 60 rpm for 5 min to dissolve the precipitate. The absorbance was read at 570 nm by a SYNERGY microplate reader (BioTek, Winooski, VT).

### Neurite outgrowth assay

Undifferentiated PC12 cells were plated in a density of 5 × 10^4^ cells/well in the above medium in 96-well plate. After cell attachment overnight each well was treated with compounds with or without 20 ng/mL NGF respectivly (NGF was dissolved in PBS, and its final volume was no more than 0.1%). After 24-h incubation, neurite outgrowth of the cells were determined by light microscopy (Leica DMI3000B, Nussloch, Germany). Neurite processes with a length equal to or greater than the diameter of the neuron cell body were scored as positive. Neurite outgrowth was determined from at least three different regions of interest in three independent experiments. Cell differentiation rate was calculated as the positive number of cells/total cell number. Data were expressed as the mean ± standard deviation (SD).

### Western blotting assay

PC12 cells were incubated at 37 °C for 24 h in a density of 1 × 10^6^ cells/well in six well palte. Then they were incubated with 20 ng/mL NGF and different concentrations of compounds for 30 min. After removal of the medium, PC12 cells were washed with ice-cold PBS, resuspended in lysis buffer containing 150 mM NaCl, 50 mM Tris (pH 8.0), 0.02% NaN_3_, 0.01% PMSF, 0.2% Aprotinin, 1% TritonX-100 supplemented with protease inhibitor cocktail (Sigma. St. Louis, Mo.), and then centrifuged at 1,2000 rmp for 15 min. The concentration of total proteins was determined using a BCA kit (Pierce, Rockford, IL). After boiling for 5 min the protein samples (30 μg) were electrophoresed on 10% SDS polyacrylamide gels and then transferred to polyvinylidene difluoride (PVDF) membrane. Nonspecific reactivity was blocked by 5% nonfat milk prepared in TBST (10 mM Tris, 150 mM NaCl, 0.05% Tween-20, pH 7.5) at room temperature for 1 h. The membranes were incubated with antibodies according to the manufacturers’ instructions. The image was captured by the Odyssey infrared imaging system (Li-Cor Bioscience, Lincoln, NE). Protein densitometry was done using Quantity One imaging software (Bio-Rad) and normalized against GAPDH.

### Statistical analysis

Data were analyzed using GraphPad Prism software (Graph Pad software Inc., San Diego, CA, USA). The comparison between two groups was analyzed by unpaired Student *t*-test, and multiple comparisons were compared by one-way ANOVA analysis of variance followed by Tukey post hoc test. Statistical significance was determined as *P* < 0.05.

## Additional Information

**How to cite this article**: Tian, X. *et al.* Distinctive effect on nerve growth factor-induced PC12 cell neurite outgrowth by two unique neolignan enantiomers from *Illicium merrillianum*. *Sci. Rep.* **5**, 16982; doi: 10.1038/srep16982 (2015).

## Supplementary Material

Supplementary Information
