# Supplementary material for: Distinctive effect on nerve growth factor-induced PC12 cell neurite outgrowth by two unique neolignan enantiomers from Illicium merrillianum
Source: Sci Rep. 2015 Nov 20;5:16982. doi: 10.1038/srep16982 (PMC4653809; doi:10.1038/srep16982)
Supplement: Supplementary Information [file srep16982-s1.pdf]

Distinctive effect on nerve growth factor-induced PC12 cell neurite outgrowth by two unique neolignan enantiomers from *Illicium merrillianum*

Xinhui Tian[1], Rongcai Yue[2], Huawu Zeng[1], Honglin Li[3], Lei Shan[1], Weiwei He[3], Yunheng Shen*[1] & Weidong Zhang*[1,2]

[1]Department of Phytochemistry, School of Pharmacy, Second Military Medical University, 325 Guohe Road, Shanghai 200433, P. R. China

[2]School of Pharmacy, Shanghai Jiao Tong University, 800 Dongchuan Road, Shanghai 200240, P. R. China

[3]School of Pharmacy, East China University of Science and Technology, 130 Meilong Road, Shanghai 200237, P. R. China

*To whom correspondence may be addressed. E-mail: wdzhangy@hotmail.com or shenyunheng@hotmail.com.

Contents

Figure S1. ESIMS, MS^2, MS^3 spectrum of compound **1**

Figure S2. ESIMS, MS^2, MS^3 fragments of compound **1**

Figure S3. HRESIMS spectrum of compound **1**

Figure S4. HPLC separation of (+)-**1** and (–)-**1** on chiral column

Figure S5. Optimized geometries of (+)-**1** (A–C) at the B3LYP/3-21G level

Figure S6. Optimized geometries of (–)-**1** (D–F) at the B3LYP/3-21G level

Figure S7. Experimental CD spectrum of (+)-**1** in methanol and the calculated ECD spectra of (7R,8S,7'S,8'S)-**1**

Figure S8. Experimental CD spectrum of (–)-**1** in methanol and the calculated ECD spectra of (7S,8R,7'R,8'R)-**1**

Figure S9. IR spectrum of compound **1**

Figure S10. ^{1}H NMR spectrum of compound **1** (500 MHz, $CDCl_3$)

Figure S11. The magnified ^{1}H NMR spectrum of compound **1** (500 MHz, $CDCl_3$)

Figure S12. The magnified ^{1}H NMR spectrum of compound **1** (500 MHz, $CDCl_3$)

Figure S13. ^{13}C NMR spectrum of compound **1** (125 MHz, $CDCl_3$)

Figure S14. DEPT NMR spectrum of compound **1** (125 MHz, $CDCl_3$)

Figure S15. ^{1}H-^{1}H COSY NMR spectrum of compound **1** (500 MHz, $CDCl_3$)

Figure S16. HSQC NMR spectrum of compound **1** (500 MHz, $CDCl_3$)

Figure S17. HMBC NMR spectrum of compound **1** (500 MHz, $CDCl_3$)

Figure S18. NOESY NMR spectrum of compound **1** (500 MHz, $CDCl_3$)

Figure S19. Single X-ray crystal structure and packing diagram of compound **1**

Crystallographic data of compound 1

Figure S1. ESIMS, MS^2, MS^3 spectrum of compound **1**

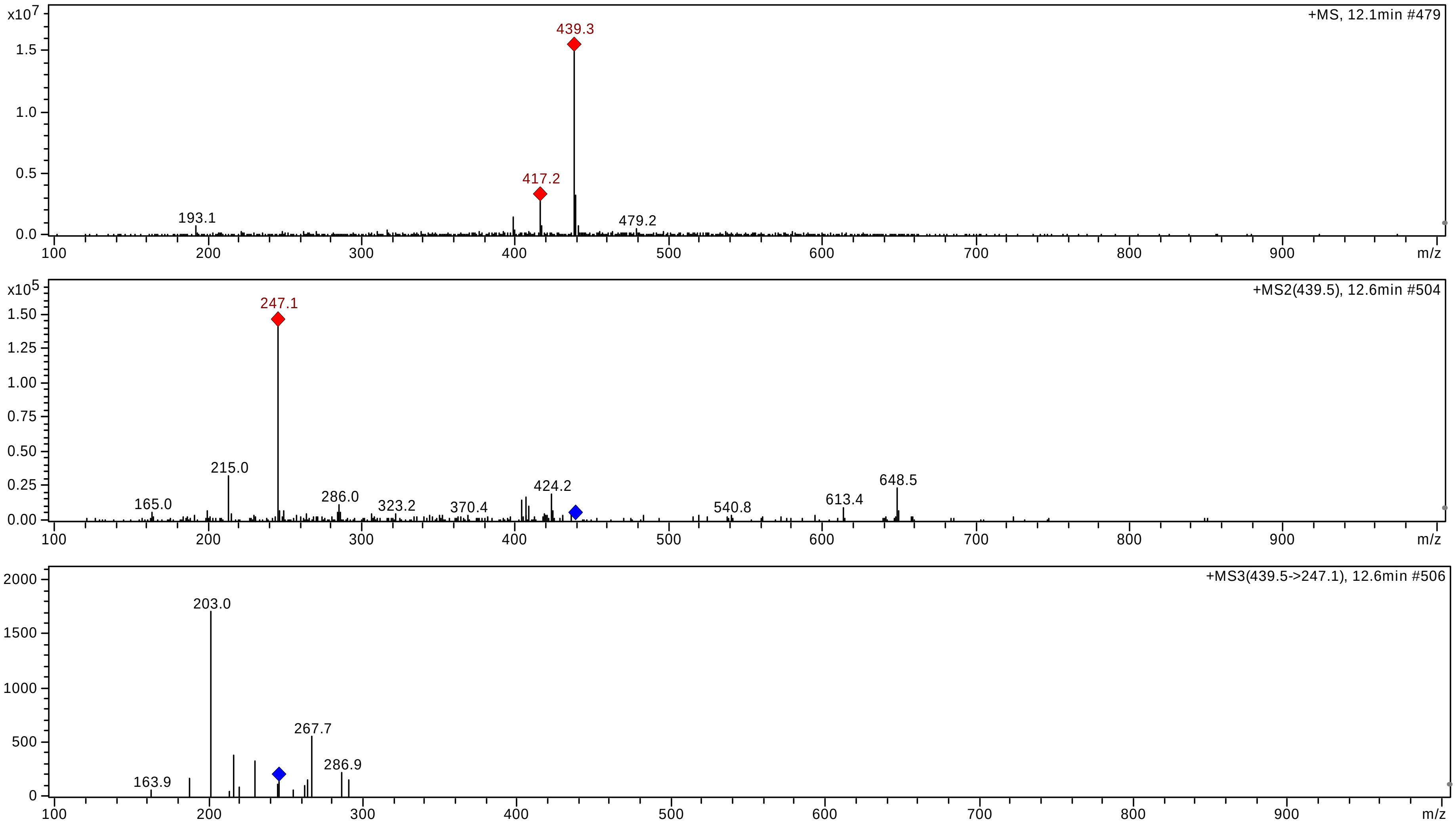

Figure S2. ESIMS, MS^2, MS^3 fragments of compound **1**

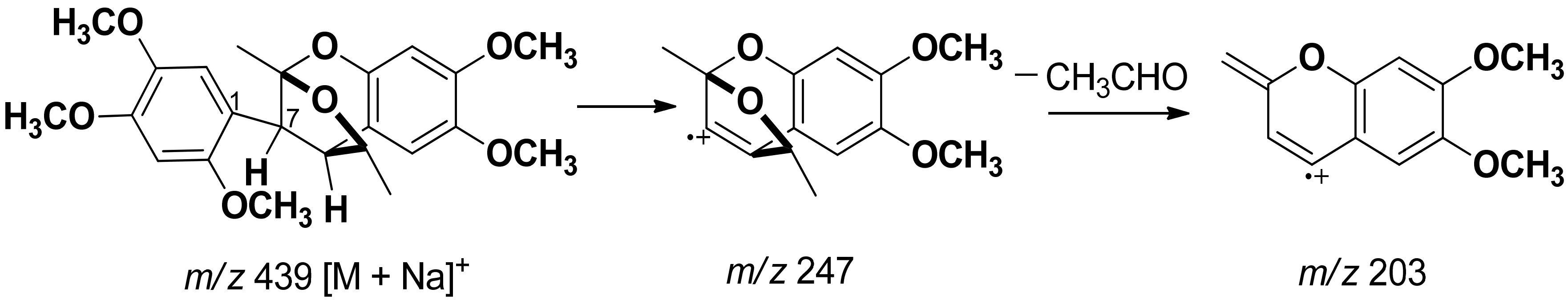

Figure S3. HRESIMS spectrum of compound **1**

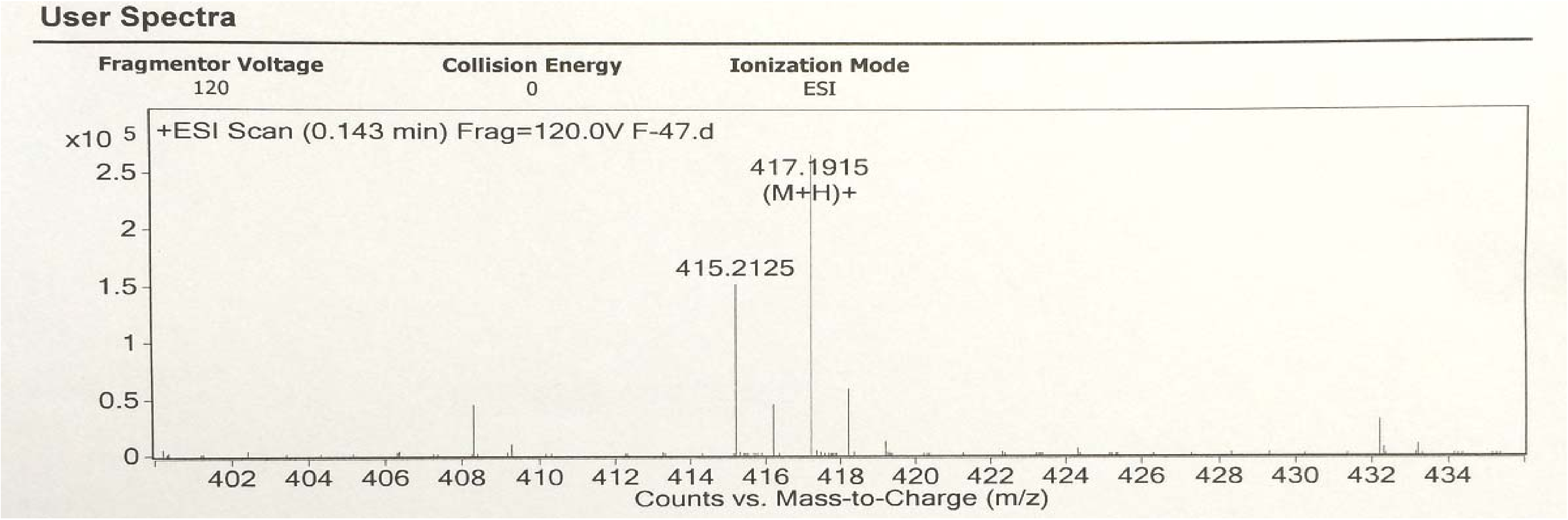

Figure S4. HPLC separation of (+)-**1** and (–)-**1** on chiral column

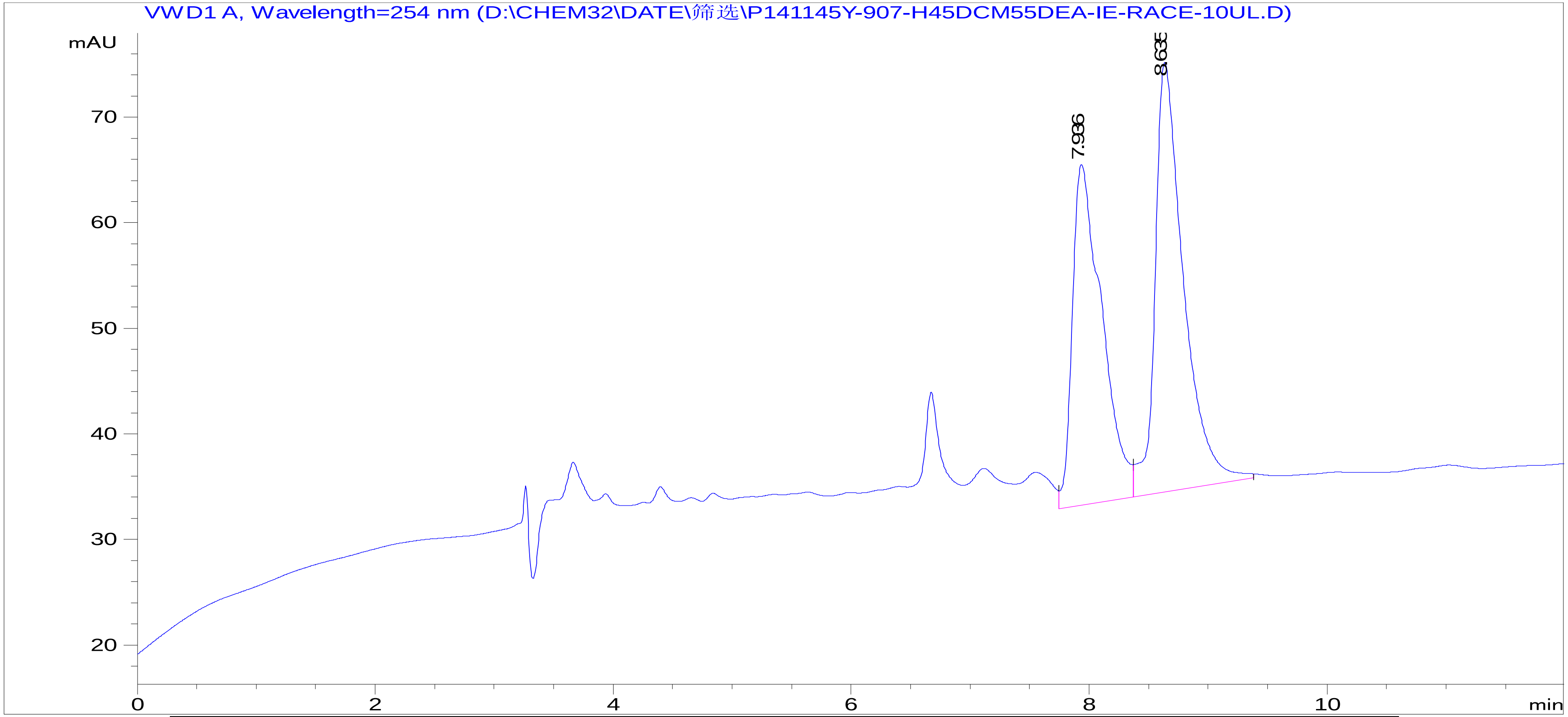

	retention time	peak area	peak height	peak width	symmetry factor	peak area %
(+)-**1**	7.935	506.5	30.8	0.2356	0.41	43.843
(–)-**1**	8.634	648.8	39.9	0.2362	0.47	56.157

Figure S5. Optimized geometries of (+)-**1** (**A**–**C**) at the B3LYP/3-21G level

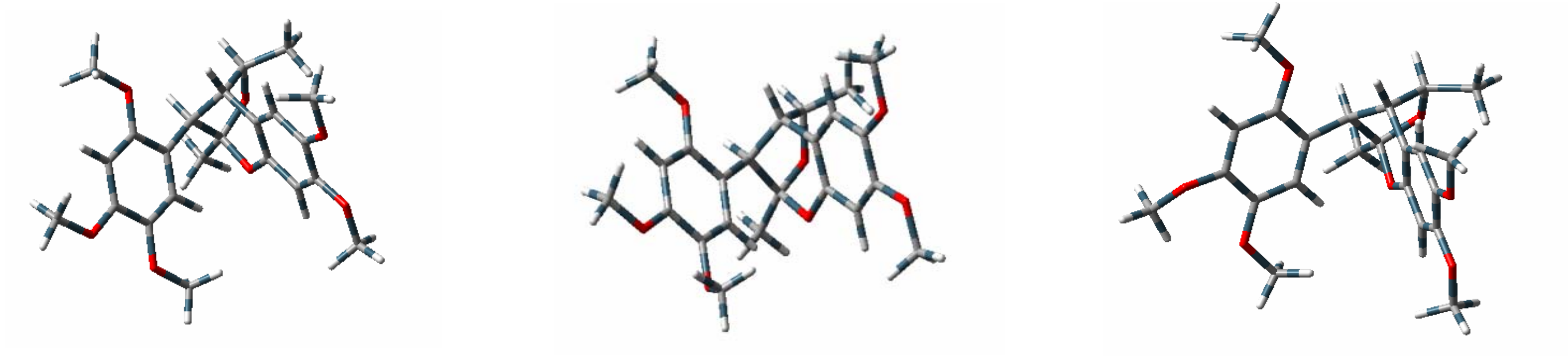

A **B** **C**

Figure S6. Optimized geometries of (−)-**1** (**D–F**) at the B3LYP/3-21G level

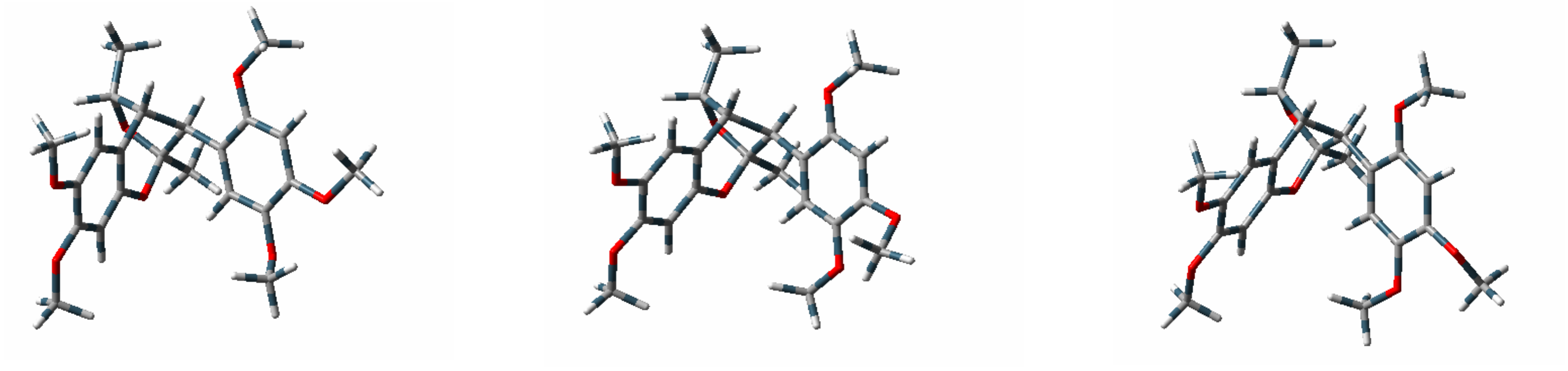

D **E** **F**

Figure S7. Experimental CD spectrum of (+)-**1** in methanol and the calculated ECD spectra of (7*R*,8*S*,7'*S*,8'*S*)-**1**

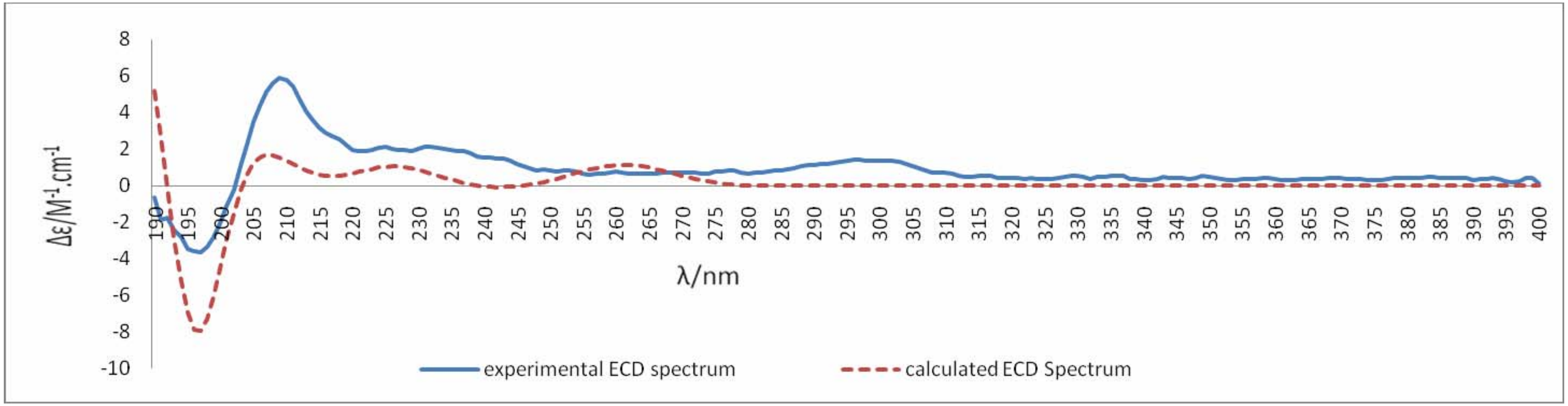

Figure S8. Experimental CD spectrum of (−)-**1** in methanol and the calculated ECD spectra of (7*S*,8*R*,7'*R*,8'*R*)-**1**

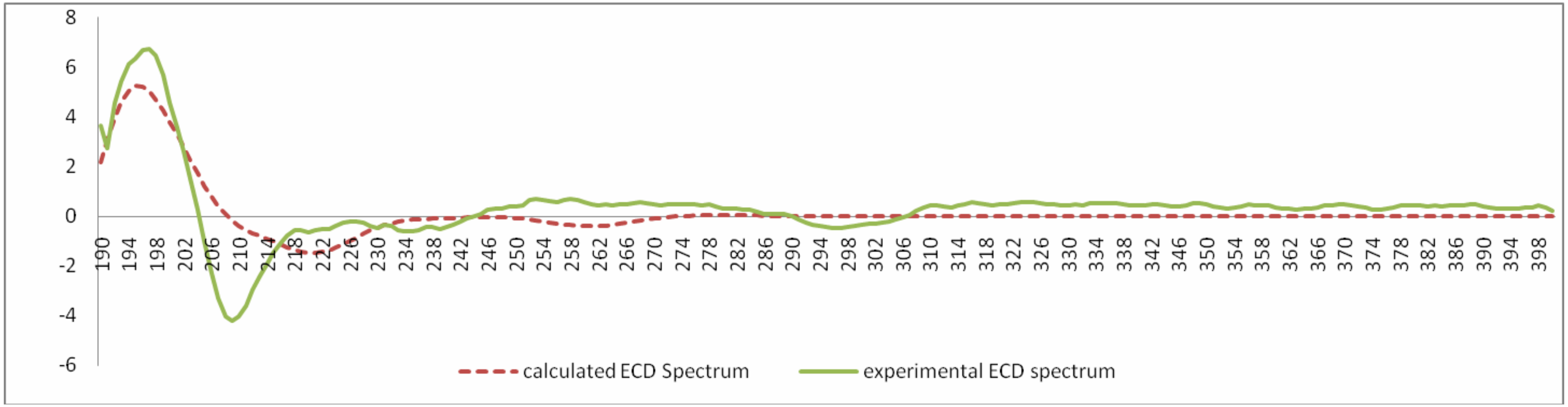

Figure S9. IR spectrum of compound **1**

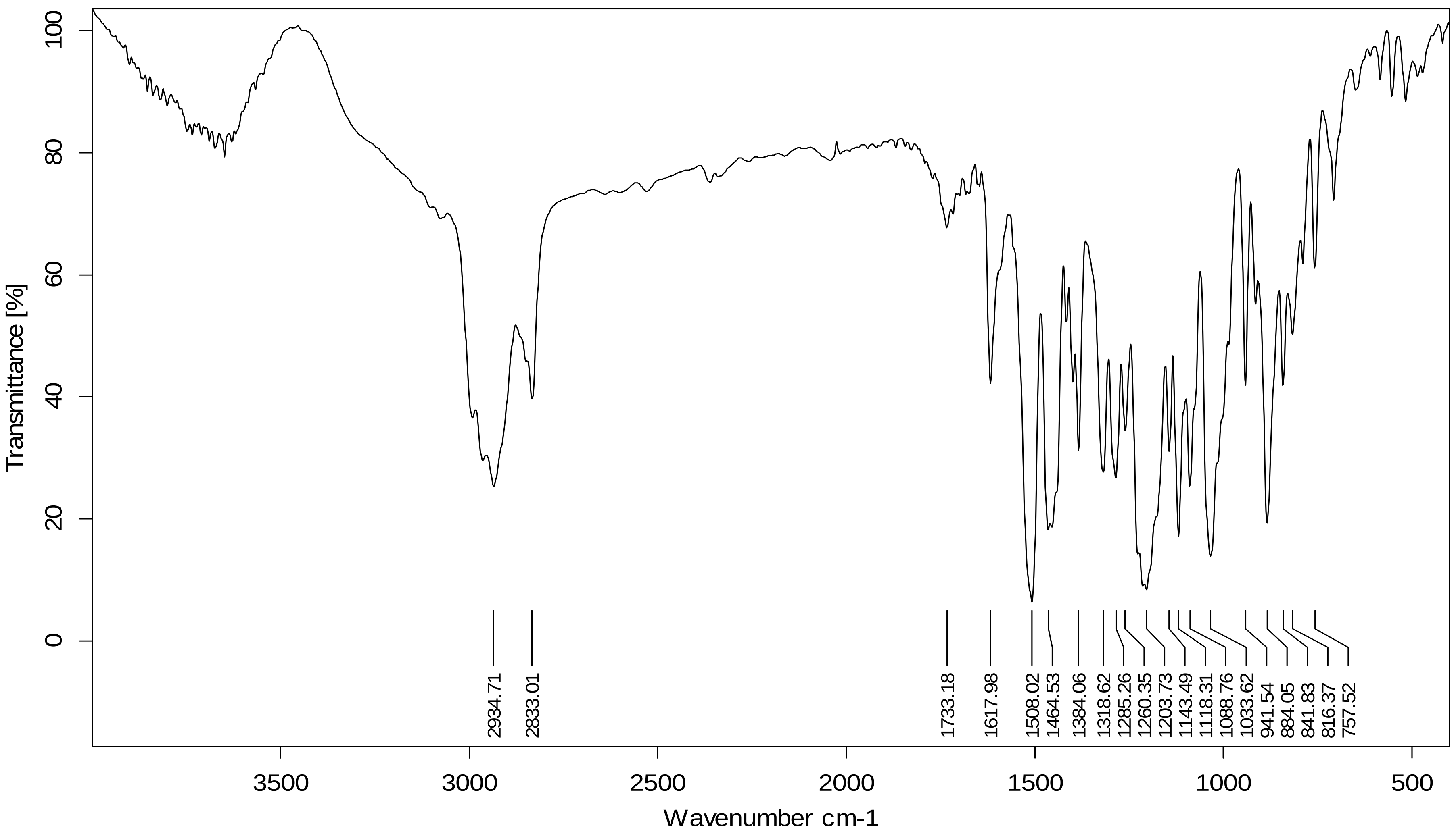

Figure S10. ^{1}H NMR spectrum of compound **1** (500 MHz, $CDCl_3$)

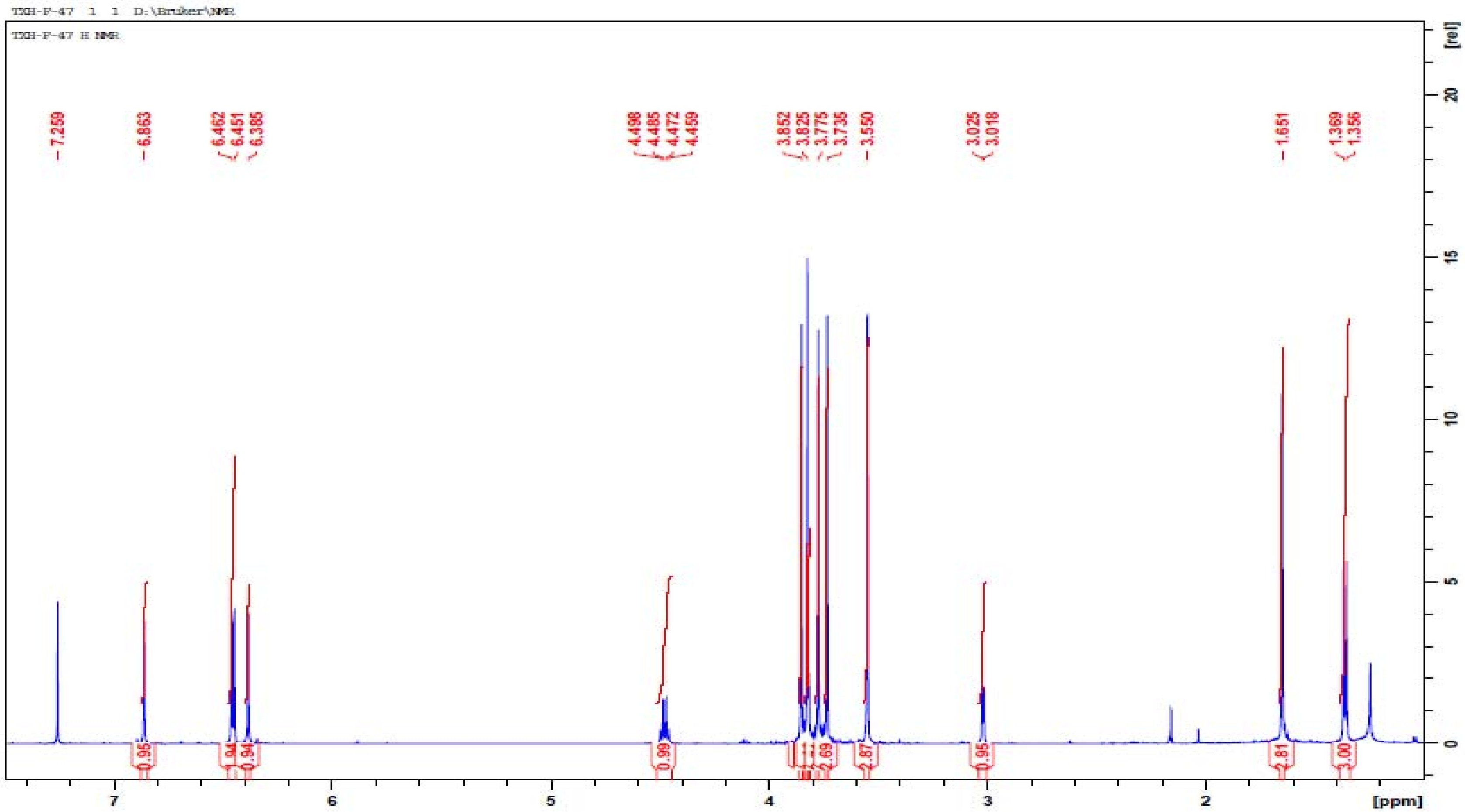

Figure S11. The magnified ^{1}H NMR spectrum of compound **1** (500 MHz, $CDCl_3$)

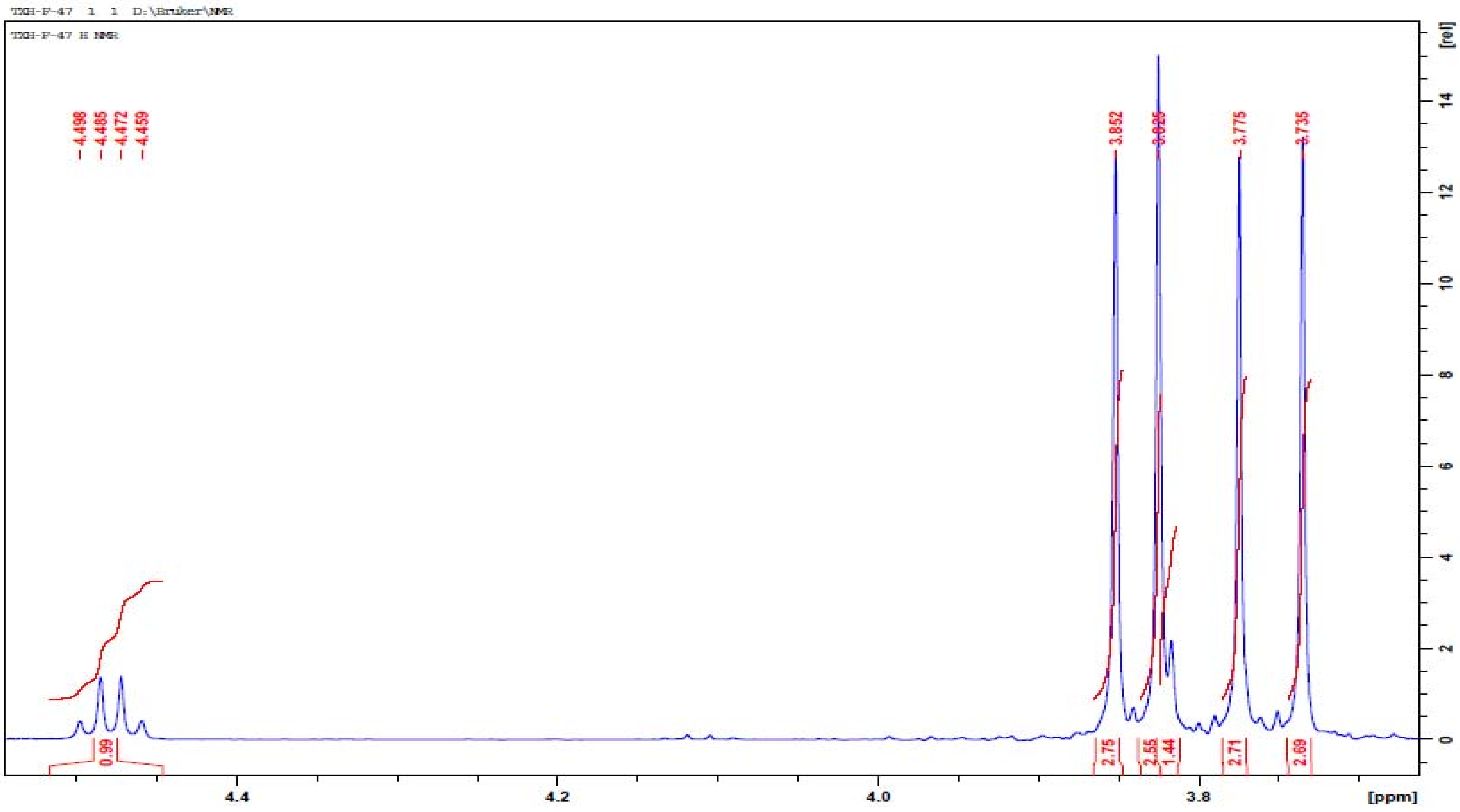

Figure S12. The magnified ^{1}H NMR spectrum of compound **1** (500 MHz, $CDCl_3$)

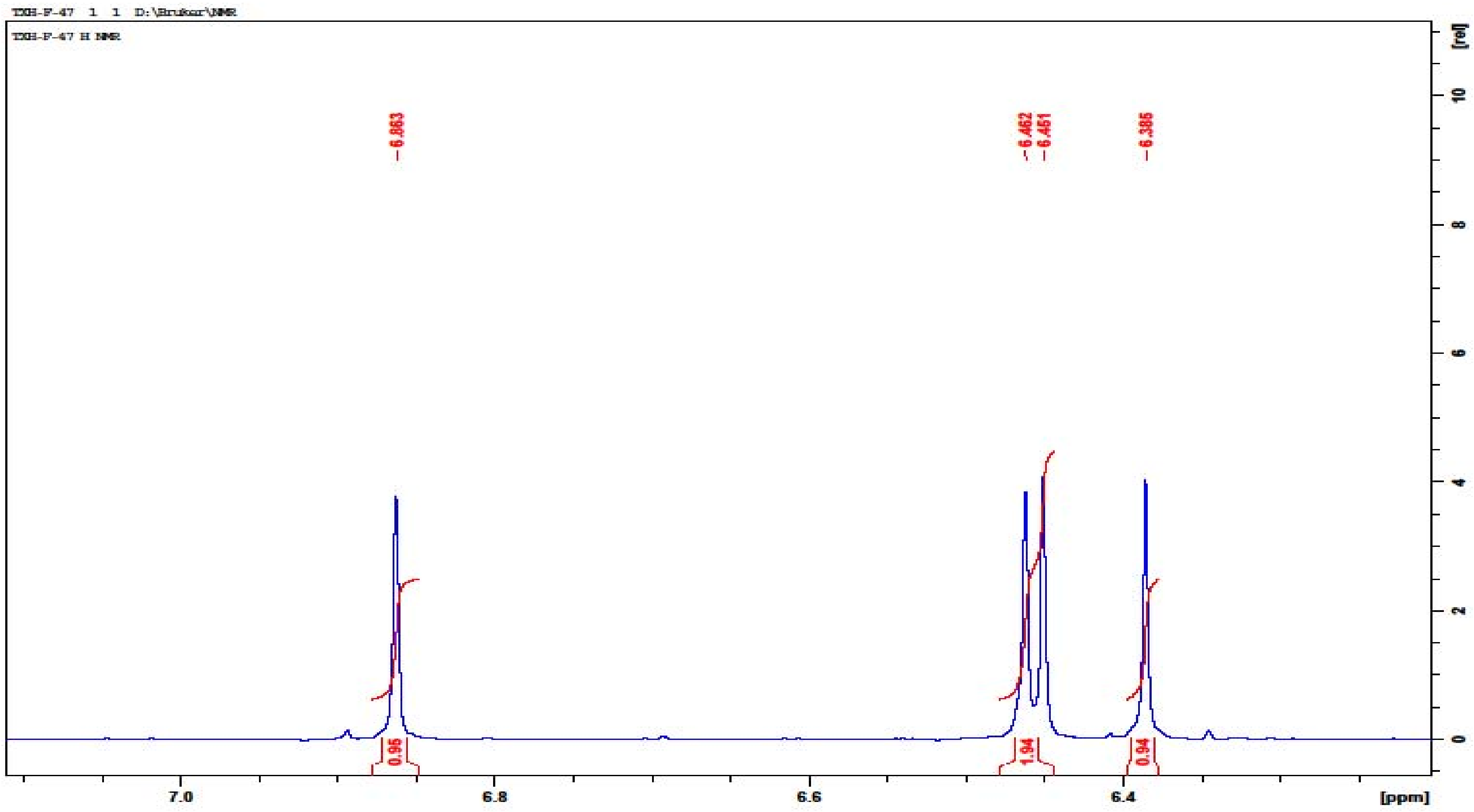

Figure S13. ^{13}C NMR spectrum of compound **1** (125 MHz, $CDCl_3$)

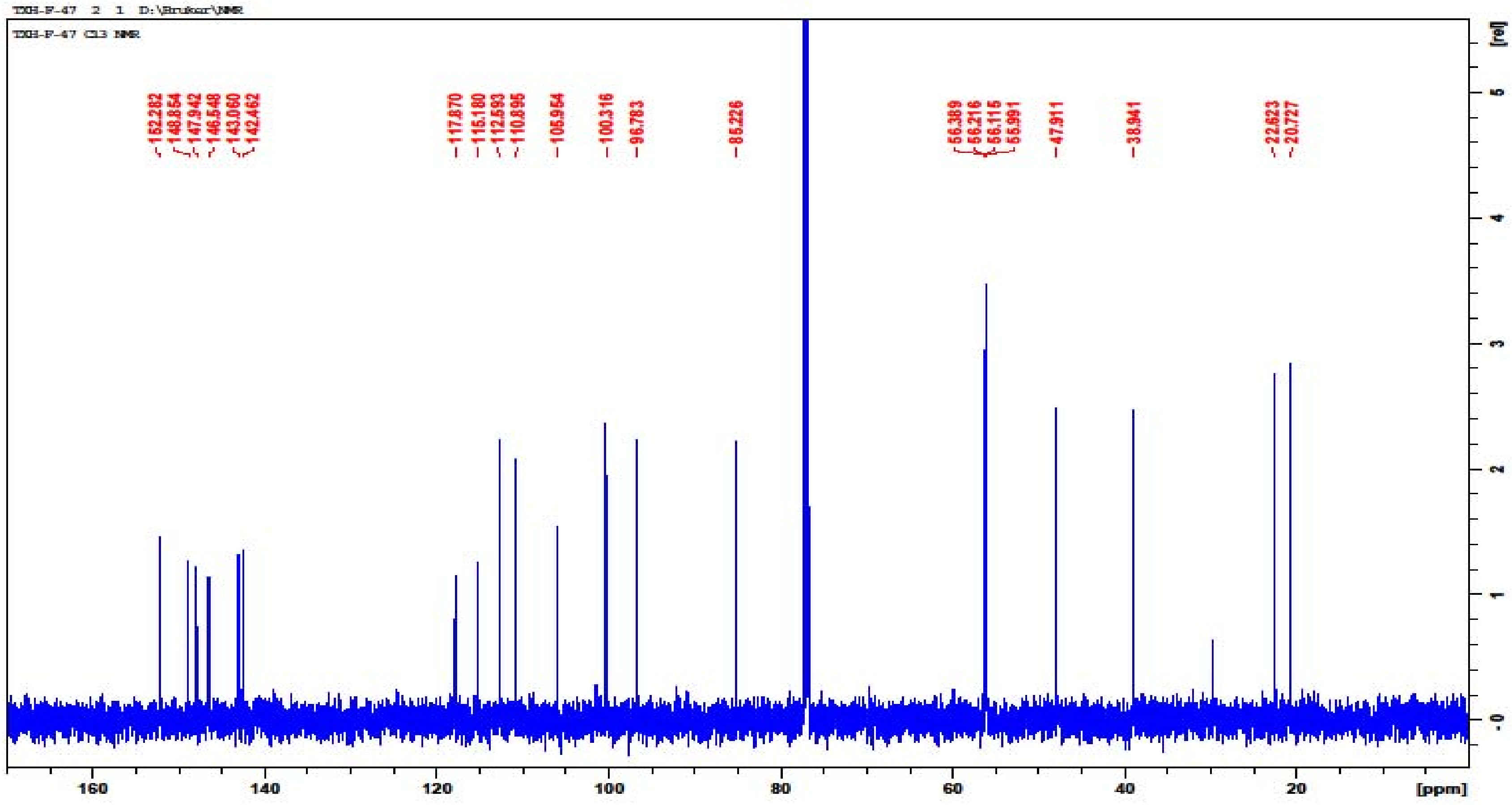

Figure S14. DEPT NMR spectrum of compound **1** (125 MHz, $CDCl_3$)

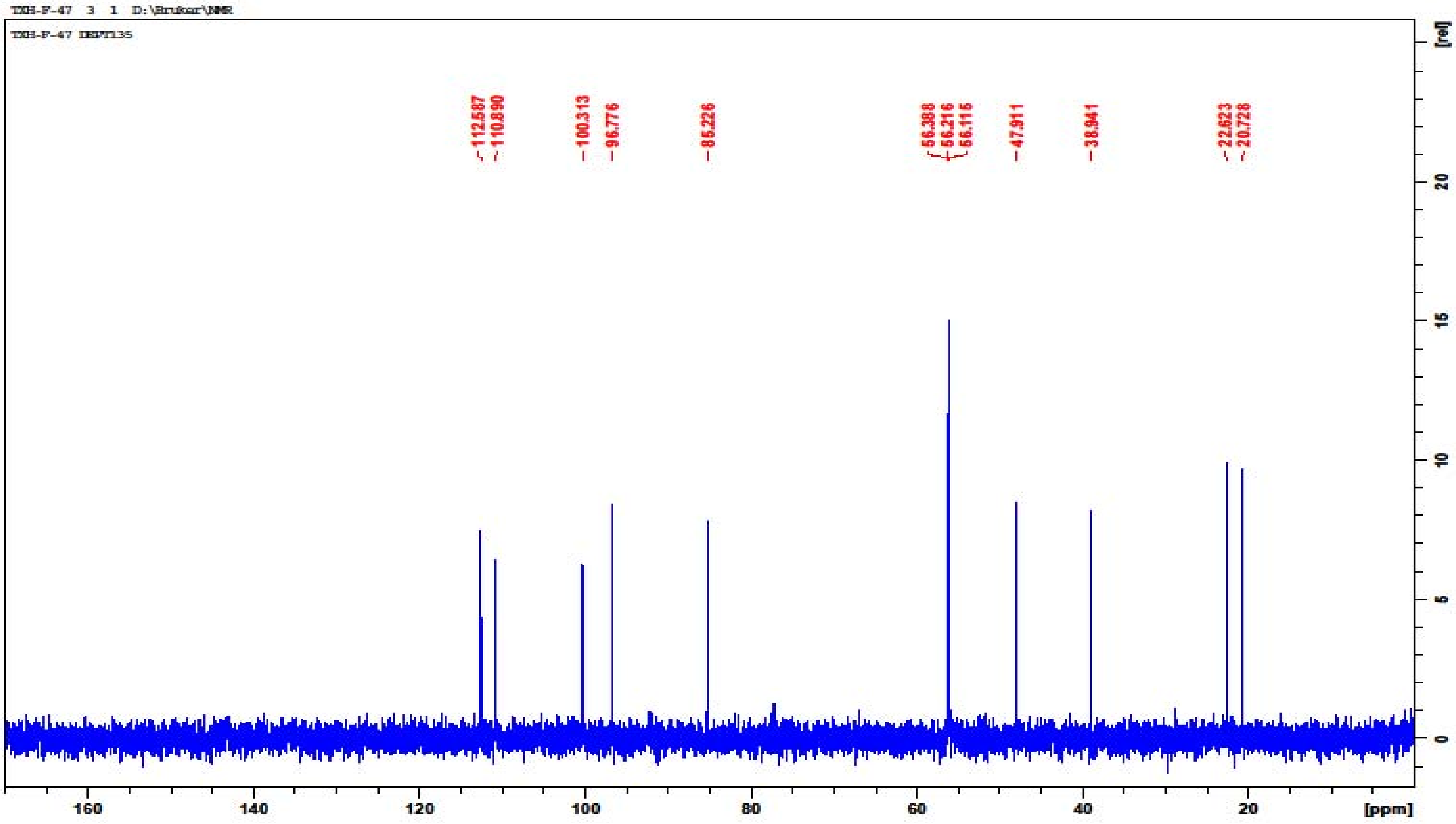

Figure S15. ^1H-^1H COSY NMR spectrum of compound **1** (500 MHz, $CDCl_3$)

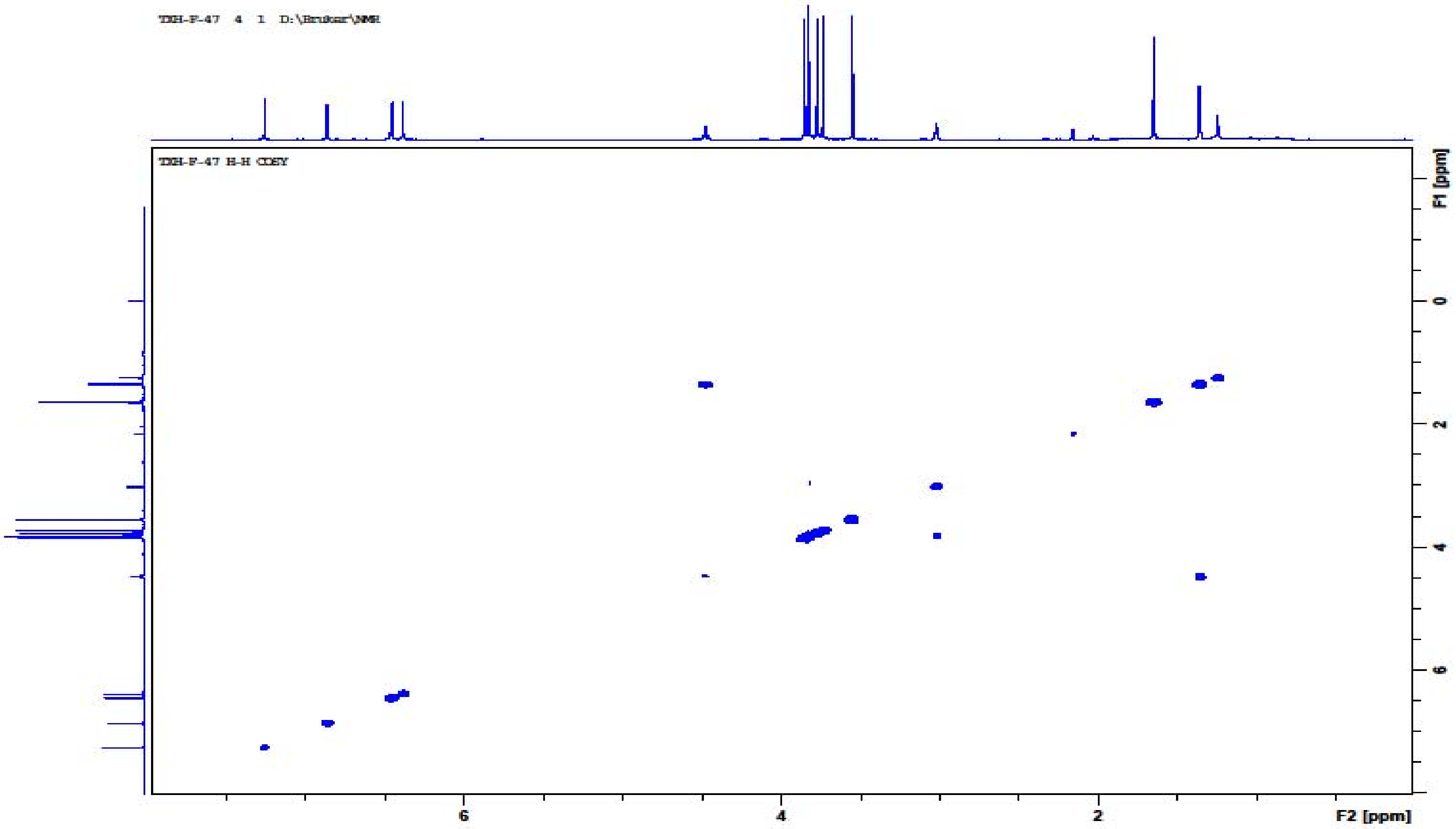

Figure S16. HSQC NMR spectrum of compound **1** (500 MHz, $CDCl_3$)

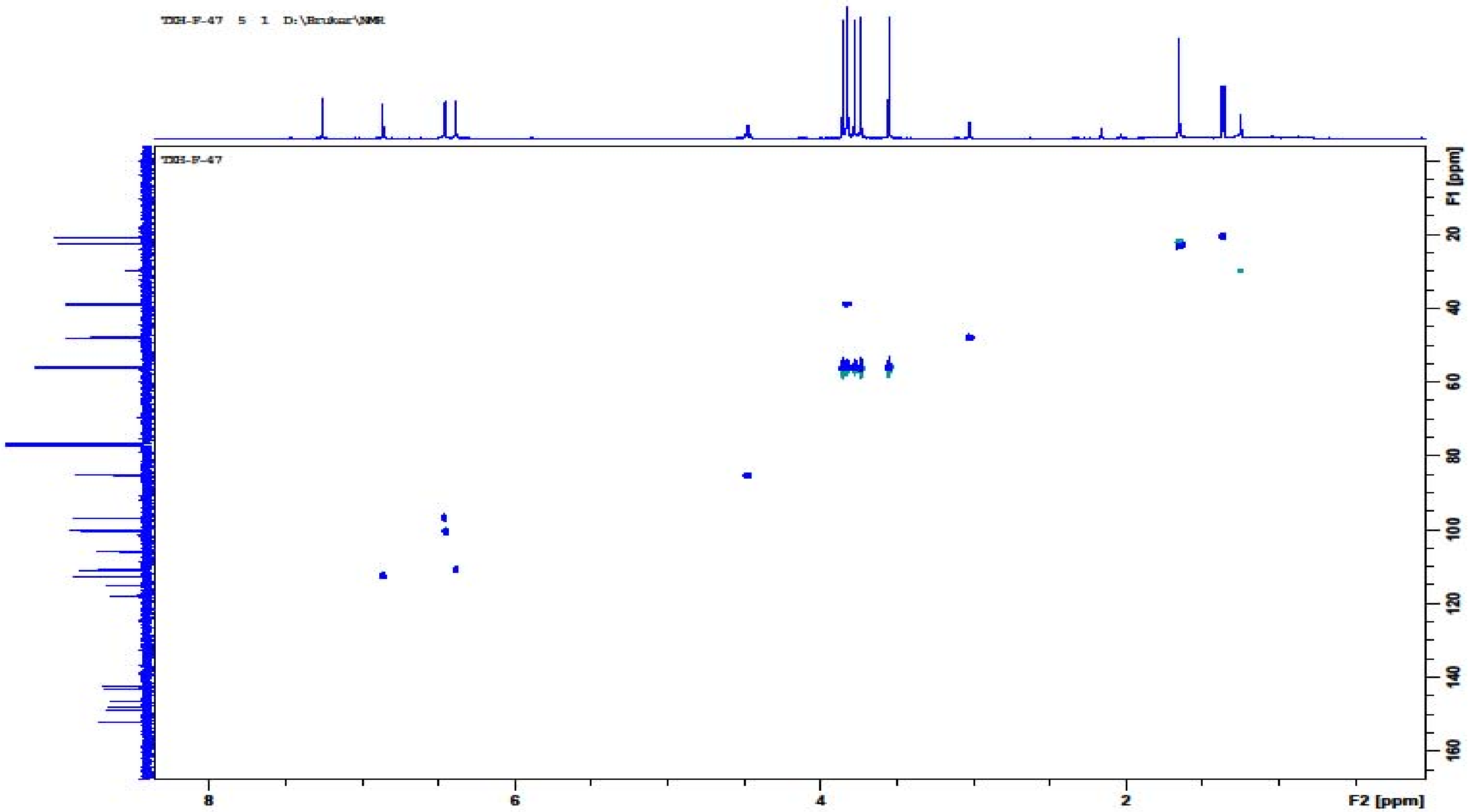

Figure S17. HMBC NMR spectrum of compound **1** (500 MHz, $CDCl_3$)

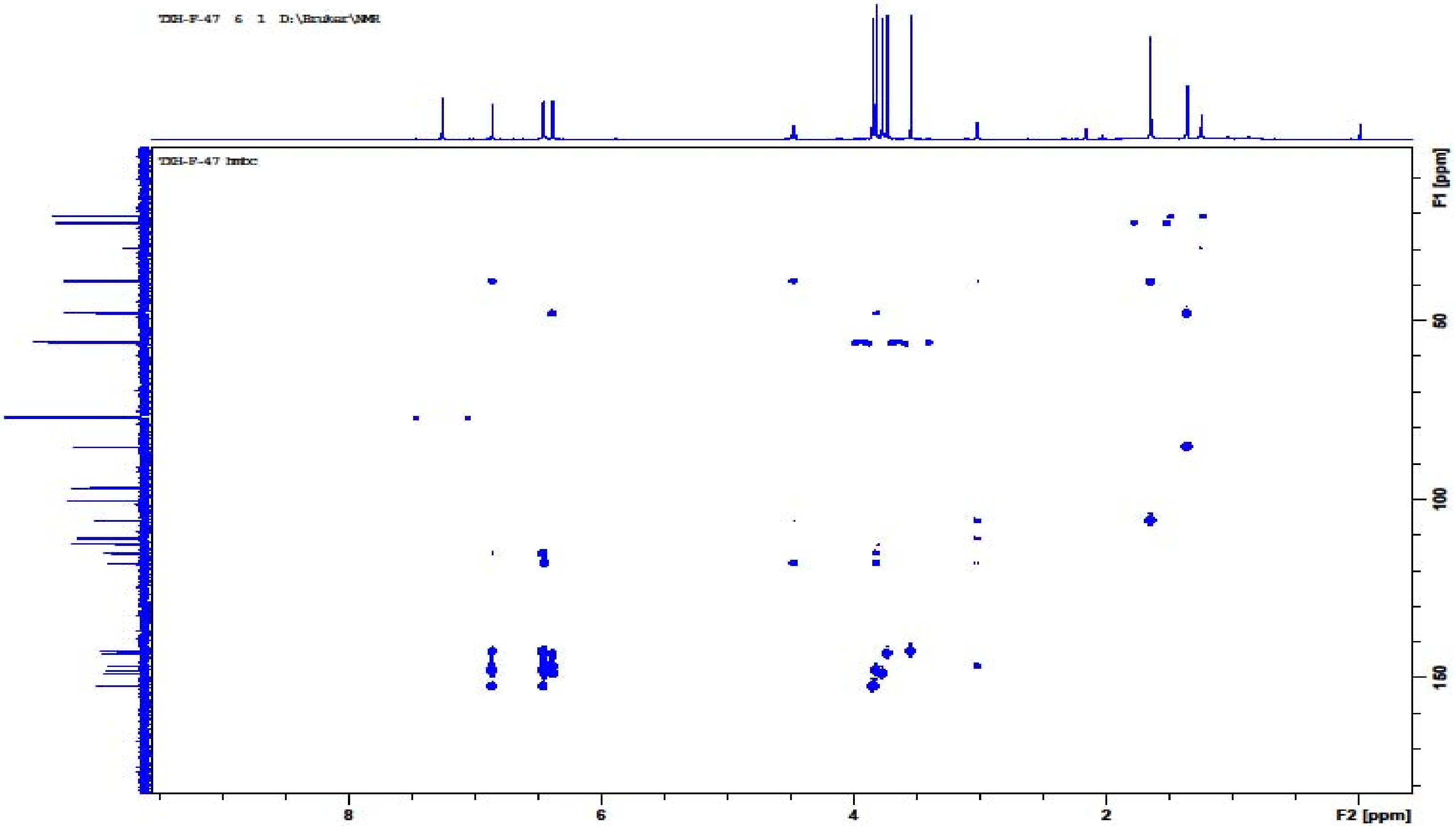

Figure S18. NOESY NMR spectrum of compound **1** (500 MHz, $CDCl_3$)

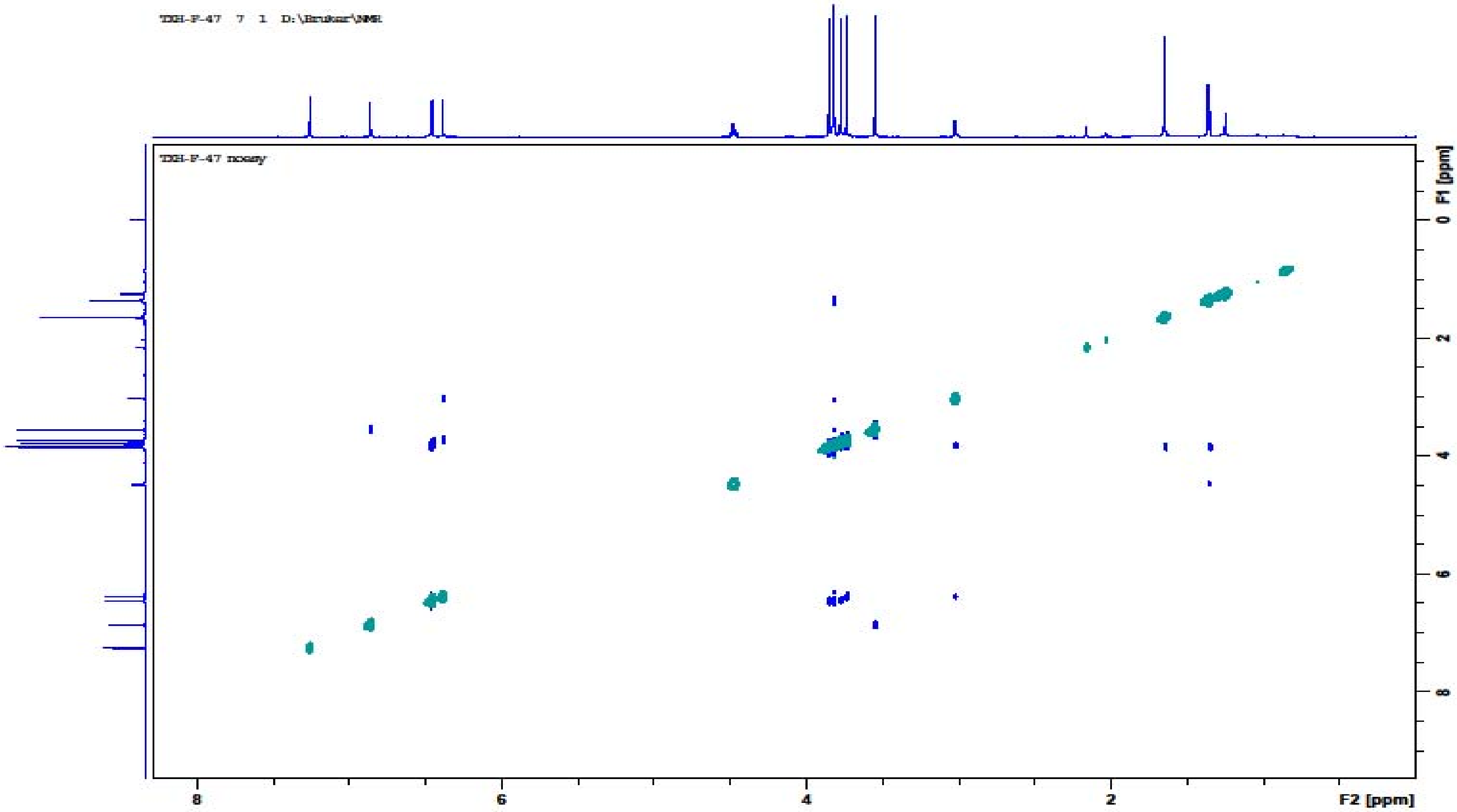

Figure S19. Single X-ray crystal structure and packing diagram of compound **1**

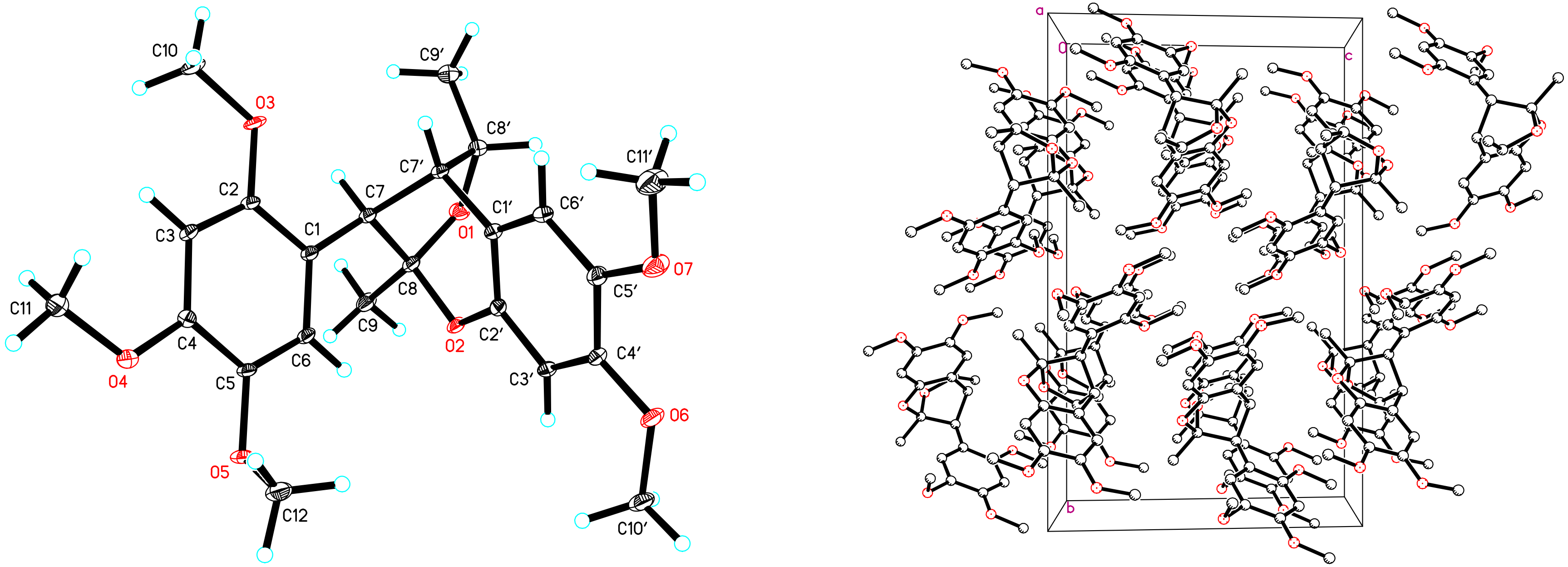

Crystallographic data of compound 1

Indentification code	cu_dm14668_0m
Empirical formula	C_{23} H_{28} O_7
Formula weight	416.45
Temperature	130(2) K
Wavelength	1.54178 Å
Crystal system	Monoclinic
Space group	P *2₁/c*
Unit cell dimensions	a = 8.94050(10) Å, α = 90°, b = 19.8567(3) Å, β = 96.5010(10)°, c = 11.89630(10) Å, γ = 90°
Volume	2098.36(4) $Å^3$
Z	4
Calculated density	1.318 Mg/m^3
Absorption coefficient	0.803 mm^{-1}
F(000)	888
Crystal size	0.20 × 0.08 × 0.03 mm^3
Theta range for data collection	4.353 to 69.420°.
Limiting indices	−10<=h<=9, −23<=k<=23, −13<=l<=11
Reflections collected / unique	10832/3816 [R(int) = 0.0316]
Completeness to theta = 67.679°	98.2 %
Absorption correction	Semi-empirical from equivalents
Max. and min. transmission	0.7532 and 0.6394
Refinement method	Full-matrix least-squares on F^2
Data / restraints / parameters	3816 / 0 / 278
Goodness-of-fit on F^2	1.037
Final R indices [I>2σ(I)]	R1 = 0.0402, wR2 = 0.1088
R indices (all data)	R1 = 0.0448, wR2 = 0.1131
Largest diff. peak and hole	0.243 and −0.257 $e.Å^{-3}$

Single crystal for analysis was obtained from MeOH solution. Data collection was performed with a *Bruker APEX2 CCD* and graphite monochromated Cu*Ka* radiation (λ = 1.54178 Å) at 130 (2) K. *Bruker* SAINT. Program used to solve and refine structure: SHELXS-97, SHELXL-97, resp. Crystallographic data for compound **1** have been deposited at the Cambridge Crystallographic Data Centre (deposition no. CCDC 1055836). Copies of these data can be obtained free of charge via www.ccdc.cam.ac.uk/conts/retrieving.html or from the Cambridge Crystallographic Data Centre, 12, Union Road, Cambridge CB21EZ, UK. [fax: (+44) 1223-336-033; or email: deposit@ccdc.cam.ac.uk].
